# Availability, appeal, and addictiveness by design: Tobacco and nicotine industry deliberate targeting of youth

**DOI:** 10.1371/journal.pmed.1005133

**Published:** 2026-05-29

**Authors:** Raglan Maddox, Becky Freeman, Charlotta Pisinger, Emily Banks

**Affiliations:** 1 Tobacco Free Program, Yardhura Walani, National Centre for Aboriginal and Torres Strait Islander Wellbeing Research, National Centre for Epidemiology and Population Health, Australian National University, Canberra, Australia; 2 School of Public Health, Faculty of Medicine and Health, The University of Sydney, Sydney, Australia; 3 Center for Clinical Research and Prevention, Bispebjerg and Frederiksberg Hospital, Copenhagen University Hospital, Copenhagen, Denmark; 4 Institute of Public Health, University of Southern Denmark, Copenhagen, Denmark; 5 National Centre for Epidemiology and Population Health, Australian National University, Canberra, Australia

## Abstract

In recognition of the World No Tobacco Day 2026 theme, "unmasking the appeal", this Perspective by Raglan Maddox and colleagues discusses how tobacco and nicotine products, particularly e-cigarettes, are deliberately designed and marketed to maximize youth appeal, and highlight the need for policies to ensure greater industry accountability and to tackle concerning uptake trends.

## Teaser

Contemporary tobacco and nicotine products, particularly e-cigarettes, are deliberately designed, marketed, and distributed to maximize youth appeal, uptake, dependence, and use. Youth uptake is a predictable outcome of systems designed to maximize product availability, appeal, and addictiveness.

## Introduction

**Fig 1:**
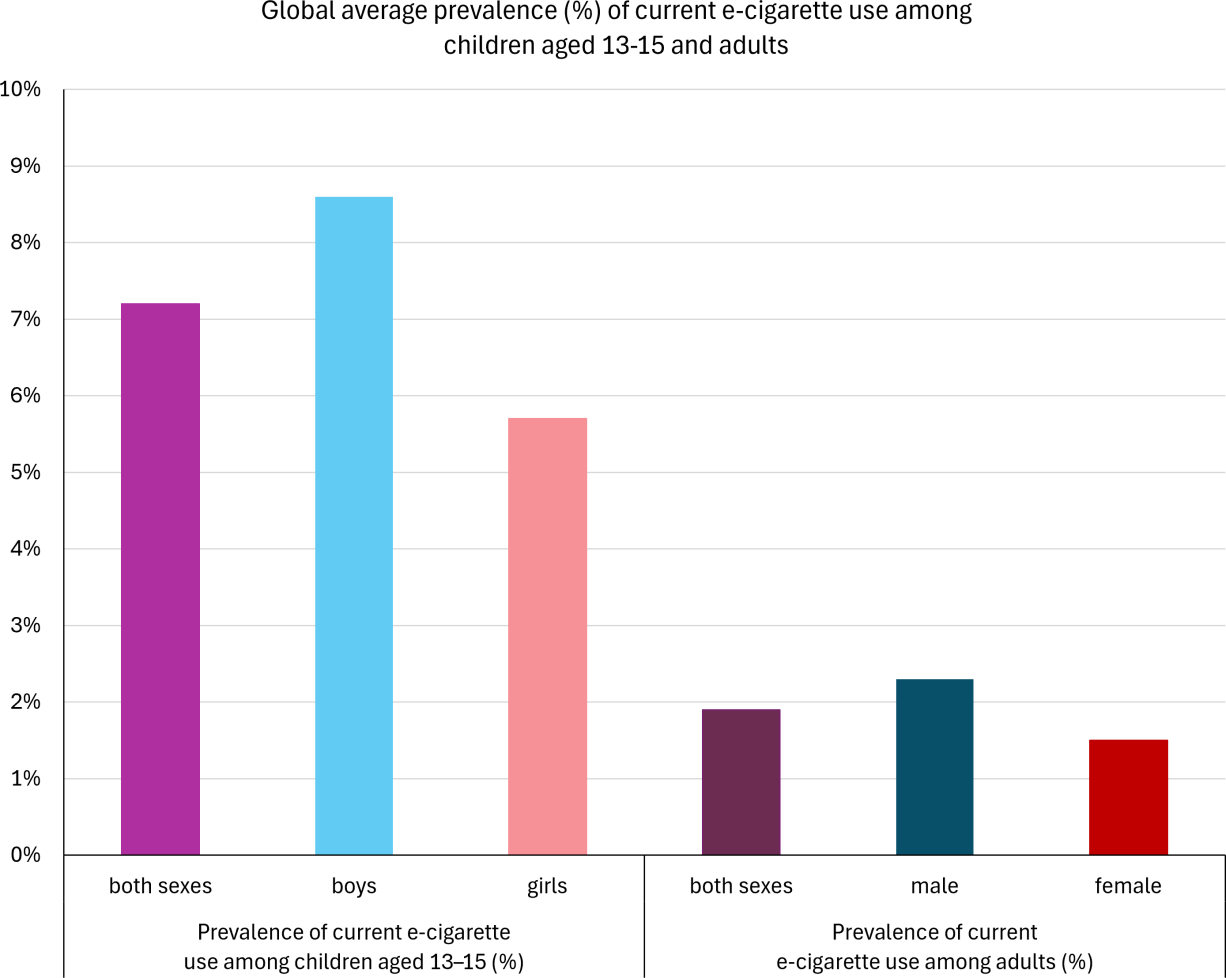
Average global prevalence of current e-cigarette use among children aged 13–15 years and adults, from countries with available data. Data adapted from WHO global estimates [1].

The 2026 World No Tobacco Day theme is “unmasking the appeal”, raising awareness of tobacco and nicotine product marketing and design, including features that increasingly appeal to adolescents. This is especially the case for e-cigarettes/vapes; vaping prevalence at ages 13–15 is on average nine times that of adults (Fig 1), and nicotine dependence among young people is increasing [1,2].

Tackling appeal is therefore imperative to reversing this trend. This means appreciating that appeal goes beyond marketing, and is deliberately built into product supply and design, market environments, and regulatory conditions that serve the structural production and sustainability aims of the tobacco and nicotine industry.

Tobacco and nicotine products intentionally attract attention, reduce aversion, and sustain use, particularly among young people who represent the next generation of consumers as smoking rates decline [3]. The commercial tobacco and nicotine industry generates profit through the creation and maintenance of addiction, and lifetime nicotine addiction largely occurs when use starts while the brain is developing, in childhood and adolescence. Youth uptake must therefore be understood not as an unintended outcome, but as a fundamental industry aim, necessary for industry profits and survival, and a predictable outcome of systems that maximize availability, appeal, and addictiveness.

Understanding these key drivers is essential for effective regulations, policies, and programs to reduce tobacco use and nicotine addiction and protect the health of young people. This is especially important given the growing evidence regarding the adverse health impacts of e-cigarettes, particularly for youth, including addiction, toxicity through inhalation, poisoning, burns and injuries, lung injury, increased smoking uptake, and concerning findings relating to cardiovascular, respiratory, and carcinogenic effects [2,4]. Furthermore, the long-term impacts of e-cigarettes on many important clinical conditions remain uncertain [2].

## The evolution of the industry narrative

Industry messaging about the purported benefits of vaping and other nicotine products has shifted from individual smoking cessation-oriented claims toward broader and often unsubstantiated narratives of population benefit that position youth uptake as incidental, unavoidable, or even protective against future smoking [5,6]. This framing relocates responsibility from industry practices onto individual behavior while preserving market legitimacy and obscuring the structural and commercial drivers of nicotine dependence [5,6].

Consistent with longstanding industry strategies to manufacture doubt and delay regulation, contemporary “harm reduction” narratives emphasize uncertainty, personal choice, and the inevitability of nicotine use, while minimizing the role of product design, marketing, and commercial systems in producing addiction [5,6]. Importantly, this framing also reproduces colonial patterns of reasoning, recasting structurally produced harms as matters of individual responsibility, which legitimizes continued expansion into younger populations [5,6]. Industry and investor communications continue to frame younger generations as critical to the long-term sustainability of nicotine markets, reinforcing the commercial importance of ongoing youth uptake and dependence.

## Why youth uptake is predictable

E-cigarette appeal arises from interacting features across product design, market environments, and regulatory conditions. These features operate synergistically, creating conditions in which initiation, dependence, and sustained use are likely [6].

At the product level, flavors [7], sensory appeal [8], device aesthetics, nicotine salts, digital integration, and misleading labeling all increase product palatability and attractiveness among young people. For example, nicotine salts allow smoother inhalation and higher nicotine delivery, accelerating dependence, particularly among people who have not previously used nicotine [8]. Devices increasingly incorporate social and digital features, embedding nicotine use within everyday routines and online environments. These features also increase visibility, peer diffusion, and social normalization among young people.

Higher nicotine concentration product use has increased substantially over time: from 2017 to 2022, the share of products containing ≥5% nicotine strength increased by 1,486% [9]. Products are marketed as long-lasting and continuously available for use, and use patterns often include ubiquitous “grazing”: repeatedly inhaling small series of puffs throughout the day. Rapid product innovation and designs intended to facilitate frequent and sustained nicotine exposure reinforce these patterns [8,9]. Products are also designed to be easy to use and integrated throughout normal daily routines and activities [8,9]. Product designs, affordability, widespread availability, and equivalence framings (e.g., puff-to-cigarette comparisons) normalize experimentation, uptake, consumption, and sustained nicotine use among young people. This pattern of continuous product change reflects a deliberate strategy to sustain use and dependence [6,9]. Misleading descriptors such as “tobacco-free” further obscure risk.

Promotion occurs within highly permissive digital and retail environments. Social media marketing, influencer partnerships, and youth-oriented branding increase exposure and normalize use at scale [10], often beyond the reach of existing regulation. These dynamics are enabled by regulatory gaps. Products frequently enter markets without robust pre-market evidence, standards remain inconsistent, and enforcement is often weak. These conditions also contribute to unregulated and illicit markets, which are a predictable consequence of oversupply, weak regulation, and profit-driven distribution [11].

Taken together, these conditions make youth uptake of products an entirely foreseeable and system-driven outcome. Importantly, exposure to appealing product environments is not evenly distributed. Youth and communities experiencing structural disadvantage are more heavily targeted and less protected by regulation. This includes Indigenous peoples, racialized populations, LGBTQA+ communities, and those facing socioeconomic disadvantage, reflecting patterns shaped by colonization, racism, and commercial targeting [12]. Describing these groups as “vulnerable” risks obscuring the structural drivers of harm [13]. The issue is not inherent susceptibility, but a failure of systems to provide adequate protection, allowing predatory industries to exploit these gaps [12].

## Policy implications: Regulating design and supply, not just marketing

Product design and other factors contributing to appeal should be recognized as key determinants of health, not as neutral features. If appeal is engineered, policy must address the systems that produce and sustain it. This requires adopting comprehensive tobacco advertising, promotion, and sponsorship bans, as well as regulating product design, supply, and the broader commercial environment [12,14].

Priorities include: pre-market product standards, where the tobacco industry is legally required to prove product safety, as in Norway; restrictions on flavors and design features (including bans); stronger control of retail and digital environments (e.g., strict tobacco retailer licensing systems, limiting retailer density, strong enforcement, and substantial penalties for violations); and full implementation of WHO Framework Convention on Tobacco Control, including high tobacco taxes, comprehensive advertising and sponsorship bans, 100% smoke-free public spaces, large graphic health warnings, restrictions on youth access, and measures to combat illicit trade [14]. This also includes Article 5.3 protections against industry interference, which recognize the fundamental conflict between public health and tobacco industry interests and require governments to protect health policy from the commercial and other vested interests of the tobacco industry through transparency, limiting interactions to those strictly necessary for regulation, and rejecting partnerships or voluntary agreements with tobacco and nicotine companies [14]. A precautionary approach is needed, placing the burden of proof of product safety on industry rather than on populations exposed to harms.

## Conclusions

Unmasking appeal requires recognizing that attractiveness is not incidental but a deliberate strategy within commercial and regulatory systems that enable this harm. Youth uptake is not a failure of individual choice or a lack of awareness of harms, but a predictable outcome of an industry structured and incentivised to generate and sustain addiction. Effective protection depends on governing commercial practices, and more fundamentally, on addressing the systems that allow harmful products to be created, promoted, and made widely available [6,11,12,14]. This shifts policy from managing risk to addressing its source: the commercial tobacco and nicotine industries.

## References

[1] World Health Organization. WHO global report on trends in prevalence of tobacco use 2000-2024 and projections 2025-2030. Geneva, Switzerland: World Health Organization; 2025.

[2] Banks E, Yazidjoglou A, Brown S, Nguyen M, Martin M, Beckwith K, et al. Electronic cigarettes and health outcomes: umbrella and systematic review of the global evidence. Med J Aust. 2023;218(6):267–75. doi: 10.5694/mja2.51890 36939271 PMC10952413

[3] Johnston M. Young smokers – prevalence, trends, implications and related demographic trends – memorandum to Dr. Robert B. Seligman. San Francisco, CA: UCSF Industry Documents Library; 1985.

[4] Golder S, Hartwell G, Barnett LM, Nash SG, Petticrew M, Glover RE. Vaping and harm in young people: umbrella review. Tob Control. 2025;:tc-2024-059219. doi: 10.1136/tc-2024-059219 40829950

[5] Maddox R, Diaz A, Waa A, Teddy L, Telford R, Pope S, et al. Resisting industry narratives: guidance to avoid tobacco and nicotine industry framing. Health Promot Int. 2024;39(6):daae143. doi: 10.1093/heapro/daae143 39569484 PMC11579600

[6] Maddox R, Heris C, Waa A, Teddy L, Upton P, Henderson PN, et al. Colonial harm in new packaging: indigenous critiques of the tobacco industry’s “harm reduction” rhetoric. Health Promot Int. 2025;40(4):daaf111. doi: 10.1093/heapro/daaf111 40728114 PMC12305302

[7] Meernik C, Baker HM, Kowitt SD, Ranney LM, Goldstein AO. Impact of non-menthol flavours in e-cigarettes on perceptions and use: an updated systematic review. BMJ Open. 2019;9(10):e031598. doi: 10.1136/bmjopen-2019-031598 31619431 PMC6797351

[8] Leventhal AM, Madden DR, Peraza N, Schiff SJ, Lebovitz L, Whitted L, et al. Effect of exposure to e-cigarettes with salt vs free-base nicotine on the appeal and sensory experience of vaping: a randomized clinical trial. JAMA Netw Open. 2021;4(1):e2032757. doi: 10.1001/jamanetworkopen.2020.32757 33433597 PMC7804919

[9] Ali FRM, Seaman EL, Crane E, Schillo B, King BA. Trends in US e-cigarette sales and prices by nicotine strength, overall and by product and flavor type, 2017–2022. Nicotine Tobacco Res. 2023;25(5):1052–6. 10.1093/ntr/ntac284 PMC10077931 36580384

[10] Lee J, Suttiratana SC, Sen I, Kong G. E-cigarette marketing on social media: a scoping review. Curr Addict Rep. 2023;10(1):29–37. doi: 10.1007/s40429-022-00463-2 41001189 PMC12459632

[11] Dessaix A, Maddox R, Stone E, Freeman B. Policy incoherence: Leadership needed to combat illicit tobacco and end tobacco oversupply. Aust N Z J Public Health. 2025;49(5):100278. doi: 10.1016/j.anzjph.2025.100278 41033930

[12] Maddox R, Bradbrook SK. Building love and justice, ending harms: a framework for abolishing the tobacco and nicotine industry. Health Promot Int. 2025;40(4):daaf103. doi: 10.1093/heapro/daaf103 40709579 PMC12290506

[13] Katz AS, Hardy B-J, Firestone M, Lofters A, Morton-Ninomiya ME. Vagueness, power and public health: use of ‘vulnerable’ in public health literature. Critical Public Health. 2019;30(5):601–11. doi: 10.1080/09581596.2019.1656800

[14] World Health Organization. World Health Organization framework convention on tobacco control. Geneva, Switzerland: World Health Organization; 2003.

